# 

**Published:** 1876-07

**Authors:** 


### ANNOUNCEMENTS FOR THE MONTH.

**MONDAYS.** SOCIETIES.

*Mondays, July* 3 and 17—Chicago Med. Society, regular meetings at the Washingtonian Home, 8 P. M.

*Mondays, July* 10 and 24 — Chicago Society of Physicians and Surgeons, regular meetings at Grand Pacific, 8 P. M.

**FRIDAYS.** SOCIETIES.

*Friday, July* 14—State Microscopical Society of Illinois, regular meeting at the Academy of Sciences, 8 P. M.

